# Simulating complex patient populations with hierarchical learning effects to support methods development for post-market surveillance

**DOI:** 10.1186/s12874-023-01913-9

**Published:** 2023-04-11

**Authors:** Sharon E. Davis, Henry Ssemaganda, Jejo D. Koola, Jialin Mao, Dax Westerman, Theodore Speroff, Usha S. Govindarajulu, Craig R. Ramsay, Art Sedrakyan, Lucila Ohno-Machado, Frederic S. Resnic, Michael E. Matheny

**Affiliations:** 1 Department of Biomedical Informatics, Vanderbilt University Medical Center, 2525 West End Ave, Suite 1475, Nashville, TN 37203 USA; 2 Comparative Effectiveness Research Institute, Lahey Hospital and Medical Center, 41 Mall Road, Burlington, MA 01803 USA; 3 UC Health Department of Biomedical Informatics, University of California San Diego, 9500 Gilman Dr. MC 0728, La Jolla, San Diego, CA 92093-0728 USA; 4 Department of Population Health Sciences, Weill Cornell Medicine, 1300 York Avenue, New York, NY 10065 USA; 5 Departments of Medicine and Biostatistics, Vanderbilt University Medical Center, 1313 21St Avenue South, Oxford House, Room 209, Nashville, TN 37232 USA; 6 Center for Biostatistics, Department of Population Health Science and Policy, Icahn School of Medicine at Mount Sinai, One Gustave L. Levy Place, Box 1077, New York, NY 10029 USA; 7 Health Services Research Unit, University of Aberdeen, Health Sciences Building, Foresterhill, 3rd Floor, Aberdeen, AB25 2ZD UK; 8 Biomedical Informatics and Data Science, Yale School of Medicine, 100 College Street, New Haven, CT 06510 USA; 9 Division of Cardiovascular Medicine and Comparative Effectiveness Research Institute, Lahey Hospital and Medical Center, Tufts University School of Medicine, 41 Burlington Mall Road, Burlington, MA 01805 USA; 10 Departments of Biomedical Informatics, Biostatistics, and Medicine, Vanderbilt University Medical Center, 2525 West End Ave, Suite 1475, Nashville, TN 37203 USA; 11 Geriatric Research Education and Clinical Care Center, Tennessee Valley Healthcare System VA, 1310 24th Avenue South, Nashville, TN 37212 USA

**Keywords:** Post-market safety surveillance, Medical devices, Learning effects, Synthetic clinical data, Simulations, Hierarchical learning effects

## Abstract

**Background:**

Validating new algorithms, such as methods to disentangle intrinsic treatment risk from risk associated with experiential learning of novel treatments, often requires knowing the ground truth for data characteristics under investigation. Since the ground truth is inaccessible in real world data, simulation studies using synthetic datasets that mimic complex clinical environments are essential. We describe and evaluate a generalizable framework for injecting hierarchical learning effects within a robust data generation process that incorporates the magnitude of intrinsic risk and accounts for known critical elements in clinical data relationships.

**Methods:**

We present a multi-step data generating process with customizable options and flexible modules to support a variety of simulation requirements. Synthetic patients with nonlinear and correlated features are assigned to provider and institution case series. The probability of treatment and outcome assignment are associated with patient features based on user definitions. Risk due to experiential learning by providers and/or institutions when novel treatments are introduced is injected at various speeds and magnitudes. To further reflect real-world complexity, users can request missing values and omitted variables. We illustrate an implementation of our method in a case study using MIMIC-III data for reference patient feature distributions.

**Results:**

Realized data characteristics in the simulated data reflected specified values. Apparent deviations in treatment effects and feature distributions, though not statistically significant, were most common in small datasets (n < 3000) and attributable to random noise and variability in estimating realized values in small samples. When learning effects were specified, synthetic datasets exhibited changes in the probability of an adverse outcomes as cases accrued for the treatment group impacted by learning and stable probabilities as cases accrued for the treatment group not affected by learning.

**Conclusions:**

Our framework extends clinical data simulation techniques beyond generation of patient features to incorporate hierarchical learning effects. This enables the complex simulation studies required to develop and rigorously test algorithms developed to disentangle treatment safety signals from the effects of experiential learning. By supporting such efforts, this work can help identify training opportunities, avoid unwarranted restriction of access to medical advances, and hasten treatment improvements.

**Supplementary Information:**

The online version contains supplementary material available at 10.1186/s12874-023-01913-9.

## Background

Data simulation provides an efficient and productive methodology for the study of theoretical foundations and novel analytic algorithms prior to their application in real world clinical data. There is an increasingly sophisticated literature regarding simulation of healthcare data to create synthetic populations with clinical features mimicking electronic health record (EHR) data [1–5]. Approaches primarily focus on replicating existing associations within EHR data to establish the validity of analyses conducted on synthetic databases [5–7] rather than on validating new methodologies. The aim of this paper is to provide a flexible, generalizable framework for synthetic data generation processes (DGPs) that can support methods development in biomedical research across clinical and medical informatics domains. We particularly focus on extending DGPs to incorporate the effects on patient safety of experiential learning at the introduction of novel medical treatments.

Medical device post-market surveillance provides a grounding use case for this effort. The U.S. Food and Drug Administration (FDA), [8, 9] Brookings Institute, [10] and major cardiovascular societies [11] have called for new sophisticated proactive approaches that can generate timely, evidence-based information to support device safety and innovation. While implantable medical devices have revolutionized healthcare over the past 50 years, device failures and post-procedural adverse events pose significant patient risk [9, 10, 12–17]. For example, conservative estimates suggest Medicare spent $1.5 billion over 10 years to replace just seven recalled models of faulty cardiovascular devices [18]. Experiential learning as providers and healthcare institutions master new technologies and treatments confers additional risk that diminishes over time, [19–31] complicating efforts to detect safety signals of new devices. Disentangling learning and intrinsic risk of a device or treatment is critical to effective post-market surveillance and safety intervention, and has been emphasized as a priority research area by FDA [32].

Correct attribution of observed risk as intrinsic to a treatment or due to learning effects can impact patient access to medical advances, guide treatment improvement, and inform training interventions. To date, however, there has been limited exploration into the detection and separation of learning effects from intrinsic risk of medical interventions. Prior research has often been retrospective, limited by small sample sizes, and/or isolated consideration of provider or institutional learning effects despite evidence of simultaneous learning at both levels [19–24, 26–31, 33–37]. These studies primarily leveraged real world data to characterize learning effects, documenting but rarely quantifying learning and not evaluating or establishing the rigor of the methods considered [33]. Fully specified simulation studies can be designed to overcome these limitations by a) providing specific challenges to new methods under development including multiple layers of learning; and b) supporting robust evaluation of new methods on complex datasets with known underlying truths.

Our DGP approach aims to support methods development and evaluation by generating synthetic data that is representative of the complex clinical environment in which novel treatments are introduced. Clinical variables important for understanding patient risk and treatment choices may be highly nonlinear, correlated with other clinical features, or influence risk through multiple interaction effects [2]. Learning may be hierarchical, occurring at both provider and institutional levels [34, 38, 39]. At both levels, learning may vary in form, speed, and magnitude [33, 39–41]. Data may be missing; adverse outcomes may be common or rare; treatment-associated risk may be strong or weak; and novel treatments of interest may vary in their adoption rate. Existing simulation methods can generate complex synthetic patient features, [1–5, 42] but are not designed to provide this full suite of specification requirements.

We developed and evaluated a novel DGP to support methods development, especially for comparative safety evaluation and learning quantification in medical device post-market safety surveillance and other clinical domains. Our DGP that provides a generalizable framework for injection of hierarchical learning effects and intrinsic treatment safety signals into complex clinical synthetic data. The DGP simulates patient features and adverse outcomes, accounting for complex feature relationships, treatment patterns, treatment safety, and multiple learning effects.

## Methods

### DGP Design

**Fig. 1:**
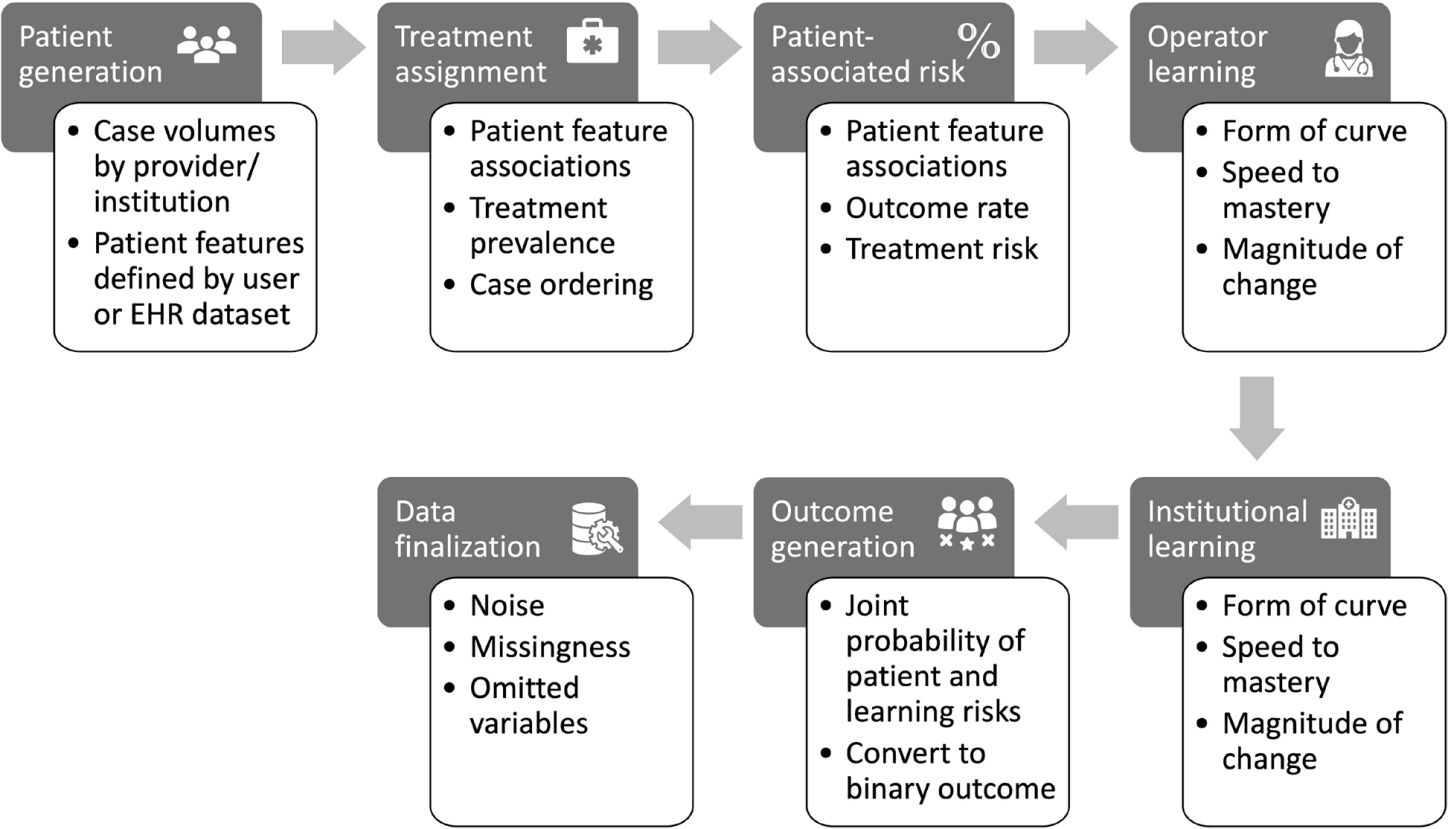
Methodologic framework of the data generating process

We designed a multi-step, hierarchical data generating process with customizable options for specification at each step (see Fig. 1 and Table 1). This design is flexible to support a variety of simulation requirements and can be applied to studies interested in multiple procedures, medical devices, or other exposures (collectively referred to as “treatments” in this paper) that may or may not be impacted by learning. The DGP first generates a synthetic patient population with patient-level features that may be specified through either defined distributions or EHR-based data cubes reflecting the complexity of data distributions and correlations. Each patient is assigned an institution, provider, and time of treatment. Treatments are assigned based on patient features, some or all of which may be associated with the probability of receiving either a novel treatment of interest or a reference treatment. Patients are sorted into treatment-specific case series that can be associated with additional risk of an adverse outcome based on provider and/or institutional experience. Outcomes are assigned based on patient risk profiles, treatment assignment, and provider/institutional learning effects. To further reflect real world complexity in clinical data, we include features to create missingness at random, omitted variables, and random noise. A full outline of the DGP process, including variable definitions and relevant equations is provided in the Appendix.

**Table 1:** Overview of available specification parameters

Parameter	Description of feature
***Patient generation***	
# of institutions	Total number of institutions to represent in the dataset
Provider distribution	Distribution of number of providers within each institution
Patient feature set	Distributions and correlations of patient features specified by EHR dataset or user definitions
Provider patient volumes	Distribution of the annual number of patients treated by a provider
Provider entry	Whether provider entry into case series should be staggered
***Treatment assignment***	
Treatment prevalence	Proportion of patients receiving each treatment
Treatment associations	Associations specifying how patient features influence treatment assignment
***Patient and treatment associated risk***	
Treatment risk	Difference in the risk of an adverse outcome associated with a novel treatment compared to the reference treatment
Outcome risk factors	Associations specifying how patient features influence the risk of an adverse outcome
Population adverse event rate	Proportion of the population experiencing an adverse outcome due to patient risk factor and treatment risk
***Provider learning-associated risk***	
Provider learning – form	Functional form of learning curve (impacts steadiness or steepness of learning)
Provider learning – speed	Number of patients receiving the novel treatment before providers reach 95% of asymptotic performance
Provider learning—magnitude	Magnitude of learning-associated risk when a provider first starts using the novel treatment
***Institutional learning-associated risk***	
Institutional learning – form	Functional form of learning curve (impacts steadiness or steepness of learning)
Institutional learning – speed	Number of patients receiving the novel treatment before institutions reach 95% of asymptotic performance
Institutional learning – magnitude	Magnitude of learning-associated risk when an institution first starts using the novel treatment
***Data finalization***	
Missingness	Proportion of missing values
Noise	Additional random noise in outcome generation
Omitted variables	Patient features to be excluded from final data

#### Patient generation

The DGP establishes the size of the patient population by first determining institution and provider patient volumes. Users specify the number of institutions; a distribution of the number of providers at each institution; a distribution of the number of patients treated annually by each provider; and the number of years to simulate. Our DGP supports bimodal distributions of both the number of providers per institution and the number of patients per provider, allowing a mixture of high and low volume providers and institutions. Each provider is assigned an entry point into their institution’s case series. For “single entry”, all providers are available to treat patients in all simulated years. For “annual entry”, half of the providers at each institution are available to treat patients in first year and the remaining providers are evenly divided to begin treating patients in each subsequent year. The DGP generates a list of providers, their assigned institution, and the number of patients they will treat in each year of the simulated series. From this list, the total sample size of patients required for the simulation is determined.

Given the established sample size, patients and their features are generated to represent the complexity of clinical patient data. We implemented Ruscio’s method for constructing synthetic populations using an iterative algorithm simulating non-normal, correlated data [43]. This approach takes as input either a) user-defined distributions and correlations; or b) clinical data cubes derived from EHRs or registries from which complex distributions and correlations can be directly estimated. This method generates categorical and continuous patient features without assumption of normality. The synthetic patient features created incorporate the complexity of the clinical data environment without including actual patient private healthcare information. Synthetic patients are randomly assigned to providers, which in turn determines their institution, and a year of treatment based on provider entry and annual patient volume.

#### Treatment assignment

Each patient is subsequently assigned to receive either a novel treatment of interest or a reference treatment. We note that the reference treatment could be an existing treatment or the decision to forgo treatment. Users specify the proportion of patients receiving the novel treatment, as well as whether and how patient features are associated with treatment choice. We implemented a logistic regression modeling framework for calculating each synthetic patient’s probability of being assigned the novel treatment. Users specify the model’s odds ratios for assignment to the novel vs reference treatment for all or select patient features. While odds ratios may be specified arbitrarily, we recommend selecting values that present a range of effect sizes that may be encountered in real world data and may highlight interesting edge cases. As such, we suggest starting with odds ratio values based on existing data or reports in in the literature and expanding possible values based on specific research questions. Patient features generated in the prior step are multiplied by the corresponding log odds ratios, and summative values are converted to the probability scale, providing each patient with a probability of receiving the novel treatment. Using a Bernoulli distribution, we convert these probabilities to treatment assignments.

Once treatments are assigned, patients are randomly shuffled within each simulated year. The resulting order defines treatment-specific case series numbers at the provider (${CN}_{prov}$) and institutional (${CN}_{inst}$) levels. For example, if a patient assigned the novel treatment had ${CN}_{prov}=1$ and ${CN}_{inst}=10$, then this would indicate the patient was the 1^st^ patient to receive the novel treatment from their provider but the 10^th^ patient to receive the novel treatment at that provider’s institution.

#### Patient and treatment associated adverse outcome probability

We calculate the probability of an adverse outcome due to patient clinical features and treatment assignment (${p}_{pt}$) based on a user-specified logistic regression model. Users provide odds ratios defining the magnitude of association between patient features and a theoretical adverse outcome. These odds ratios may include associations with all or select patient features. Users also specify the odds ratio for an adverse outcome associated with the novel treatment, indicating the relative increase or decrease in probability associated with the novel treatment compared to the reference treatment. As with the odds ratios for treatment assignment, we recommend estimating values from existing data or as reported in the literature, adjusting as needed to create more challenging simulations of interest. An adverse outcome rate for the population must be specified and determines the intercept of the logistic model when applied to the synthetic patient population.

Patient features and treatment assignment are multiplied by the corresponding log odds ratios, and summative values are converted to the probability scale, providing ${p}_{pt}$ for each patient observation. This is the independent probability of an adverse outcome due solely to patient features and treatment.

#### Provider learning-associated adverse outcome probability

Any treatment (novel and/or reference) may have some probability of an adverse outcome associated with provider learning ($${p}_{prov})$$. The magnitude of this probability is based on each patient’s treatment-specific provider-level case series number ($${CN}_{prov}$$), with risk declining to 0 as cases/experience accumulate [44]. For each treatment, users specify the initial magnitude of learning-associated risk (i.e., maximum $${p}_{prov}$$ which occurs when $${CN}_{prov}=1$$); how many cases are required for providers to achieve mastery of the treatment (i.e., probability due to learning diminishes to 0); and a functional form for the change in probability over a case series. In accordance with prior learning curve research [33, 39, 45], our DGP supports a variety of functional forms. Currently implemented forms are monotonically decreasing, thus learning-associated probability of an adverse event diminishes as case number and experience increase. We implemented learning curve forms from the power, exponential, reciprocal, and Weibull distributions (see Appendix Figure A.1 for illustrative examples of each). We note, however, users may specify alternative curve functions. As an example, the following formula estimates the provider learning-associated probability of an adverse outcome under exponential learning:

$${p}_{prov}={b}_{{0}^{prov}}*\mathrm{exp}\left(-{b}_{{1}^{prov}}*{CN}_{prov}\right)$$

Form-specific parameters, ${b}_{{0}^{prov}}$ and ${b}_{{1}^{prov}}$ above, are calculated within the DGP process based on user-specified magnitude of learning and number of cases required for the learning effect to diminish to 0. When provider-level learning effects are not specified for a particular treatment, the probability of an adverse outcome due provider learning is defined as ${p}_{prov}=0$ for all observations assigned to the treatment.

#### Institutional learning-associated adverse outcome probability

As with provider-associated learning, any treatment may have some risk associated with institutional learning ($${p}_{inst})$$. This probability is calculated in a manner parallel to that of provider-associated learning. For each treatment, users specify the initial magnitude of learning-associated risk (i.e., maximum $${p}_{inst}$$ which occurs when $${CN}_{inst}=1$$); how many cases are required for institutions to achieve mastery of the treatment; and a functional form for the change in probability over a case series. These values are independent of those specified at the provider level and may be included in the presence or absence of provider learning. The same functional forms are available at the provider and institutional levels, though users may specify different forms at both levels. For example, above we provided the equation for exponential learning and here we provide an example of institutional learning-associated probability of an adverse outcome with a power form:

$${p}_{inst}={b}_{{0}^{inst}}* {\left({CN}_{inst}\right)}^{-{b}_{{1}^{inst}}}$$

Form-specific parameters, ${b}_{{0}^{inst}}$ and ${b}_{{1}^{inst}}$ above, are calculated within the DGP process based on user-specified magnitude of learning and number of cases required for the learning effect to diminish to 0. When institution-level learning effects are not specified for a particular treatment, the probability of an adverse outcome due institutional learning is defined as ${p}_{inst}=0$ for all observations assigned to the treatment.

#### Outcome generation

We defined adverse outcomes as resulting from the confluence of independent risks due to patient characteristics and learning at each level. Independence is a simplifying assumption of the simulation process compared to real-world data, see the Discussion section for an examination of implications and alternatives. For each observation, the DGP calculates the probability of an adverse outcome by combining independent probabilities of an adverse event due to 1) patient and treatment associated risk (${p}_{pt}$); 2) provider learning-associated risk (${p}_{prov}$); and 3) institutional learning-associated risk (${p}_{inst}$). As we are interested in the overall probability of an adverse event resulting from the union of these independent factors, we combine them using the joint probability of not experiencing an event due to any of the three components as:

$${p}_{noevent}=\left(1-{p}_{pt}\right)*\left(1-{p}_{prov}\right)*\left(1-{p}_{inst}\right)$$

We convert back to the overall probability of an adverse event as:

$${p}_{event}=\left(1-{p}_{noevent}\right)$$

Using a Bernoulli distribution, we convert these probabilities to binary outcomes.

#### Data finalization

At this stage, we have a complete synthetic dataset incorporating learning effects with patient and treatment risks factors. To support a range of simulation objectives, we provide optional steps to incorporate additional challenges that may impact methods used in biomedical research. Medical data may have variable signal-to-noise ratios, suffer from missing data, include patient features of unknown relevance, and omit information on important patient features. The DGP includes the option to add noise by randomly substituting non-adverse events with adverse events and vice versa for a specified proportion of patients. Random patient feature values can optionally be masked to create missingness at random, and users can request the DGP drop a set of patient features from the final datasets by specifying omitted variables.

#### Code availability

We have made our DGP publicly available at https://tinyurl.com/2zre28zs. We provide an R script containing functions for each step of the data generation process, as well as a script following an example workflow for generating a simulated dataset.

### DGP Evaluation

To illustrate the alignment of our synthetic datasets with user specifications, we conducted a simulation study across diverse combinations of DGP parameters. We generated datasets with 5–30 institutions, split evenly into large (10 provider) and small (5 provider) institutions. Providers were assigned to be high or low volume, with 20–30 or 5–15 cases per year, respectively. Case series were simulated over either 2 or 4 years. We did not predefine the exact number of observations to be generated for each dataset. Rather, the combination of number of institutions, number of providers per institution, volume of cases assigned to each provider, and number of years in the case series determined the number of observations generated for each dataset at the time of simulation. This led to a wide distribution of sample sizes for consideration and emphasized small and moderate samples over large datasets.

As a basis for patient feature distributions and correlations, we constructed an EHR-based data cubed with 35 patient-level features using publicly-available MIMIC-III data [46] and definitions from a prior study of indwelling arterial catheters in these data [47]. The population adverse outcome rate was set to 2%, 5%, or 10%. We assigned each patient to one of two potential treatments, mimicking the situation where an established treatment existed on the market and another novel treatment was recently FDA approved. We specified three levels of novel treatment prevalence (10%, 25%, and 50%) and treatment-associated risk (odds ratios of 1.0, 1.5, and 2.0). Provider and institutional learning effects were either absent, present for one level but not the other, or present at both levels. When specified, we simulated power, exponential, or Weibull learning curves. The initial magnitude of provider-associated learning, when present, was set at 20–60% of the mean probability of an adverse outcome due to patient features and treatment assignment among patients assigned to the relevant treatment. The learning effect declined to 0 over the provider’s first 10 or 25 cases. The initial magnitude of institution-associated learning, when present, was set at 5–25% of the mean probability of an adverse outcome due to patient features and treatment assignment among patients assigned to the relevant treatment. The learning effect declined to 0 over the institution’s first 100 or 200 cases.

There were 38,988 unique combinations of these parameter settings. One dataset was generated for each unique combination. Full specification details are available in the Appendix.

We validated the synthetic datasets against specifications by estimating key parameters from the realized data. We evaluated similarity of patient feature distributions between the synthetic populations and the reference MIMIC-III cohort by comparing variable means, medians, and variability. We compared the median and variance of continuous patient features in synthetic data to the MIMIC-III data using the Wilcoxon rank sum test [48] and Levene's test of homogeneity, [49] respectively. Binary and categorical features were compared with chi-squared tests. From each synthetic dataset, we estimated realized population outcome rates (prior to the incorporation of any learning), novel treatment prevalence, and novel treatment odds ratio. Realized novel treatment odds ratios were estimated from correctly parameterized logistic regression models incorporating patient features and treatment assignment. Deviations between realized and specified parameter settings were examined overall and across sample size to determine whether there was a lower bound for generating simulations responsive to specifications.

## Results

**Fig. 2:**
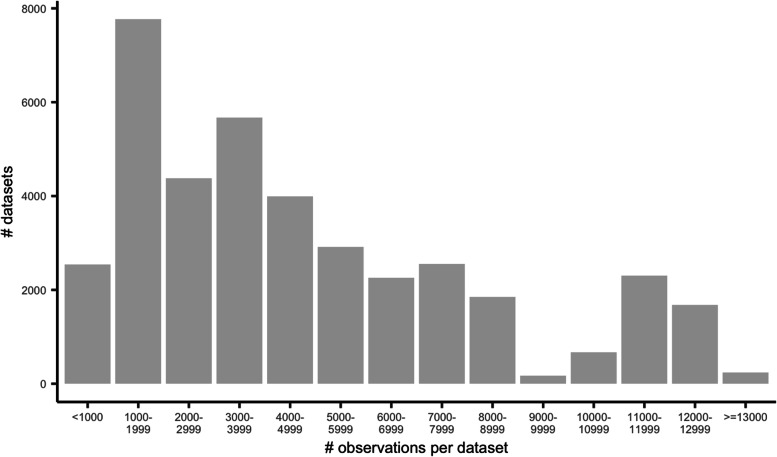
Distribution of the number of simulated observations in each dataset

**Fig. 3:**
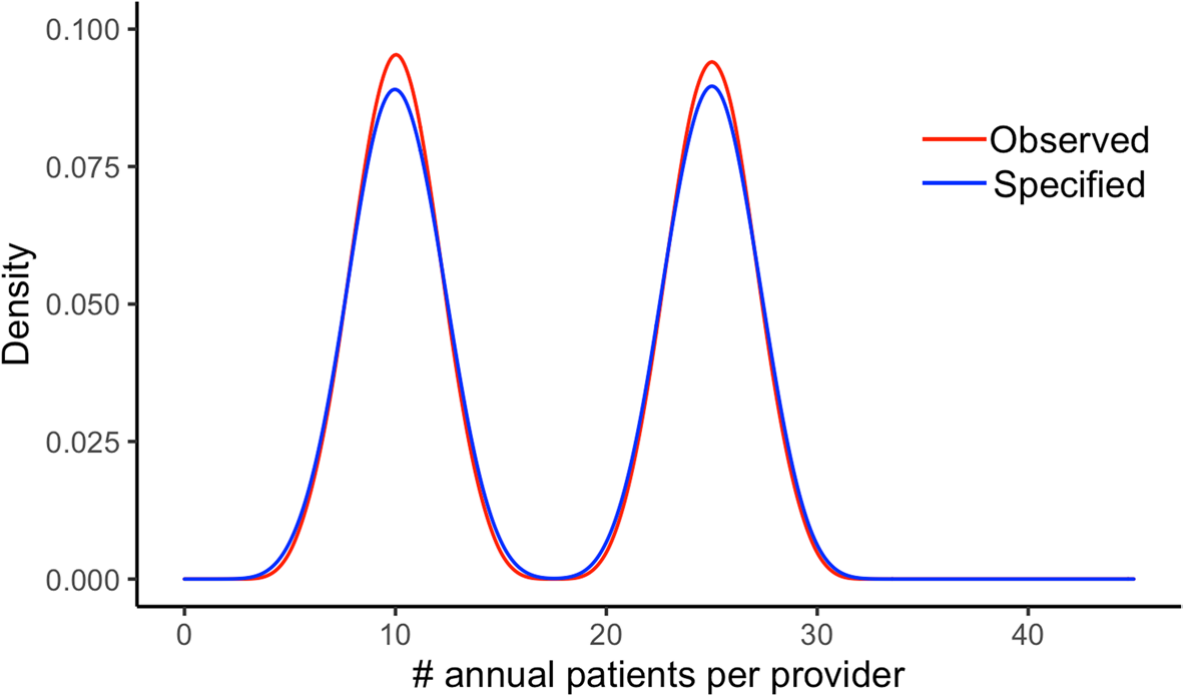
Distribution of annual case volumes by provider

Among all synthetic datasets simulated, the number of patients ranged from 559 to 14,690 with a mean of 4,798. The distribution of sample sizes generated across the 38,988 datasets is presented in Fig. 2. Figure 3 highlights agreement between the specified and realized mix of high and low volume providers among 1,000 randomly selected synthetic datasets.

**Fig. 4:**
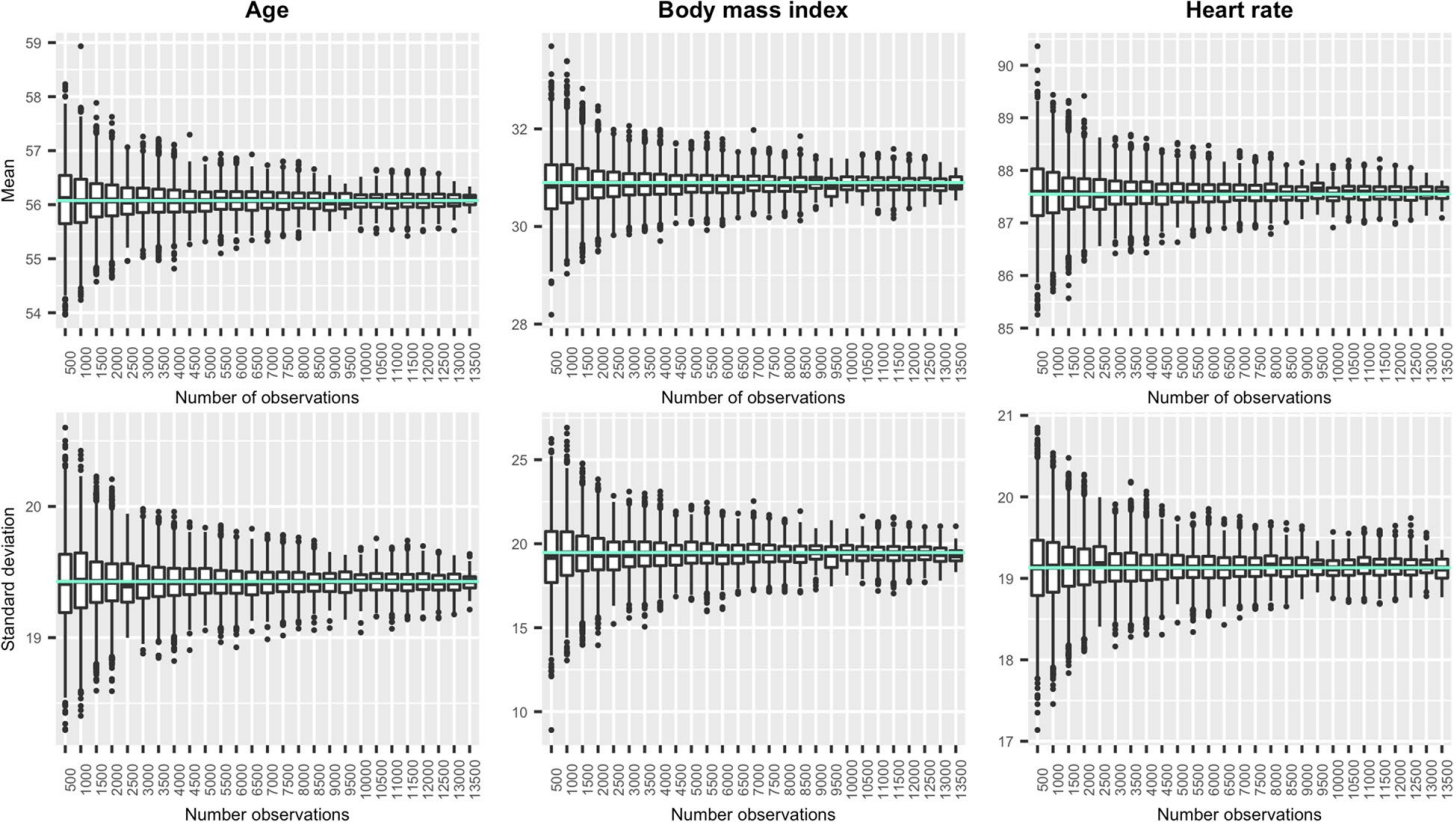
Distributions of simulated patient features by sample size. Mean and variance of select patient features by simulated sample size compared to reference value in underlying MIMIC data (blue line)

We observed agreement between the distributions of patient features in the synthetic data compared to the reference MIMIC-III dataset from which patient characteristics were derived. Figure 4 displays the central tendency (mean) and variability (standard deviation) of age, body mass index, and heart rate in synthetic datasets by sample size (see Appendix for details of all patient features). Across datasets, mean and variance reflected the underlying reference population, with no significant differences after adjustment for multiple comparisons. Deviations from reference population values, while relatively small in all cases, were largest when generating small datasets. For example, the mean age in the MIMIC-III reference population was 56.1 years. Among datasets with < 1000 versus datasets with > 10,000 observations, the mean age ranged from 53.7 – 58.9 years and 55.4 – 56.8 years, respectively. The mean body mass index in the MIMIC-III data was 30.9. Mean body mass index for the same sample size groups ranged from 28.8 – 33.6 and 30.2 – 31.7, respectively. On average, reference population means were within the 95% confidence interval 94.8% of the time, with no trend by sample size. Variability in both the mean and standard deviation of feature distributions declined as sample size increased, with improvement slowing as sample sizes grew beyond 3,000 observations. As we leveraged an existing, previously validated method for generating patient features from a reference EHR-based data cube, additional comparisons of simulated and reference values are provided in the Appendix.

We observed strong agreement between specified and realized treatment and outcome parameters across all generated sample sizes. For each level of the novel treatment prevalence, the mean and median values were equivalent to the specified value across the range of sample sizes. Treatment prevalence ranged from 7.3–13.3% when specified at 10%, from 21.0–29.1% when specified at 25% and 45.4–55.3% when specified at 50%. For each level of the adverse outcome rate, the mean and median values were equivalent to the specified value across the range of sample sizes. Outcome rates ranged from 1.0%-3.6% when specified at 2%, from 3.2–7.6% when specified at 5% and 7.0%-13.6% when specified at 10%.

**Fig. 5:**
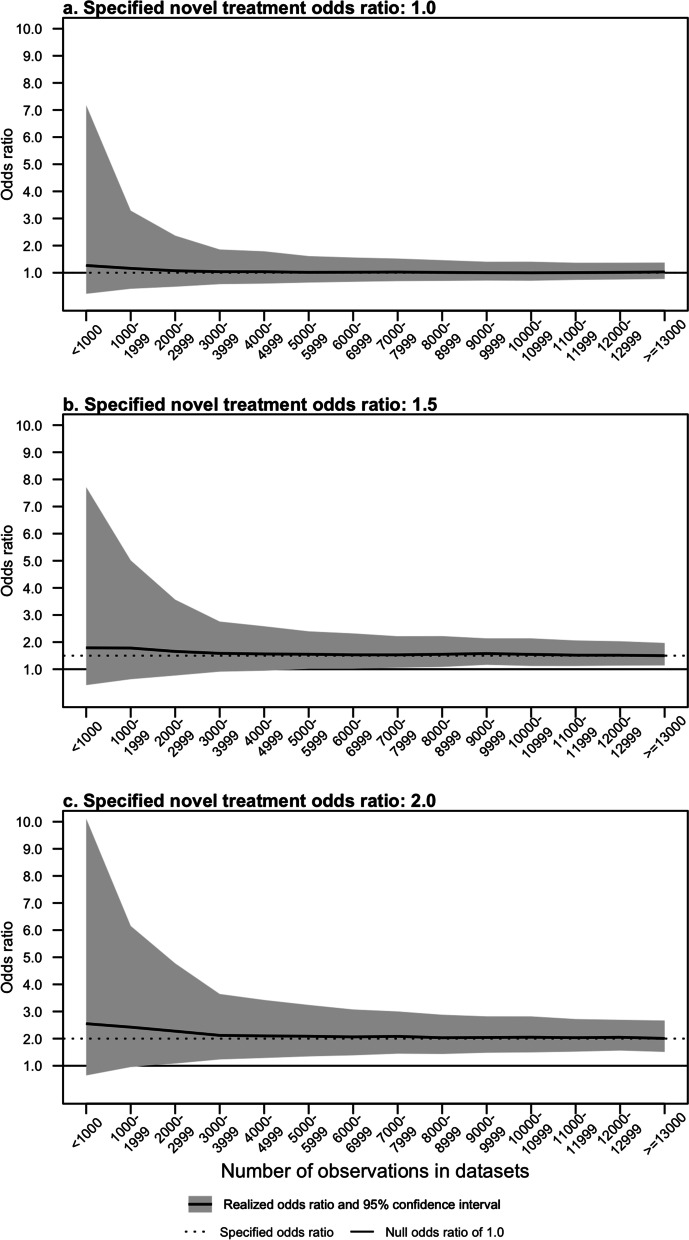
Estimated novel treatment odds ratios and confidence intervals by sample size and specified value

Agreement between specified and estimated realized effect of the novel treatment varied by simulated sample size. Figure 5 illustrates how estimates and confidence intervals of novel treatment odds ratios change across the range of simulated sample size. For datasets with the smallest sample sizes, estimated realized treatment effects overestimated the specified value and confidence intervals were wide. We note that although point estimates of the odds ratio were high in the smallest datasets, confidence intervals for the novel treatment effect captured the specified value in 94.1% of simulated datasets across all sample sizes. Estimates converged to the specified value as sample sizes reached approximately 3,000–5,000. Confidence intervals correctly excluded 1.0 when the novel treatment signal was specified to be above this null value when sample sizes exceeded 5,000 and 1,000 when the specified odds ratios were 1.5 and 2.0, respectively. We note that one must be careful interpreting these findings as an indication that the simulated datasets did not reflect specified treatment effects in small datasets, as recovering odds ratios by estimating values in a small population are subject to high uncertainty even if the true population value approaches the specification.

**Fig. 6:**
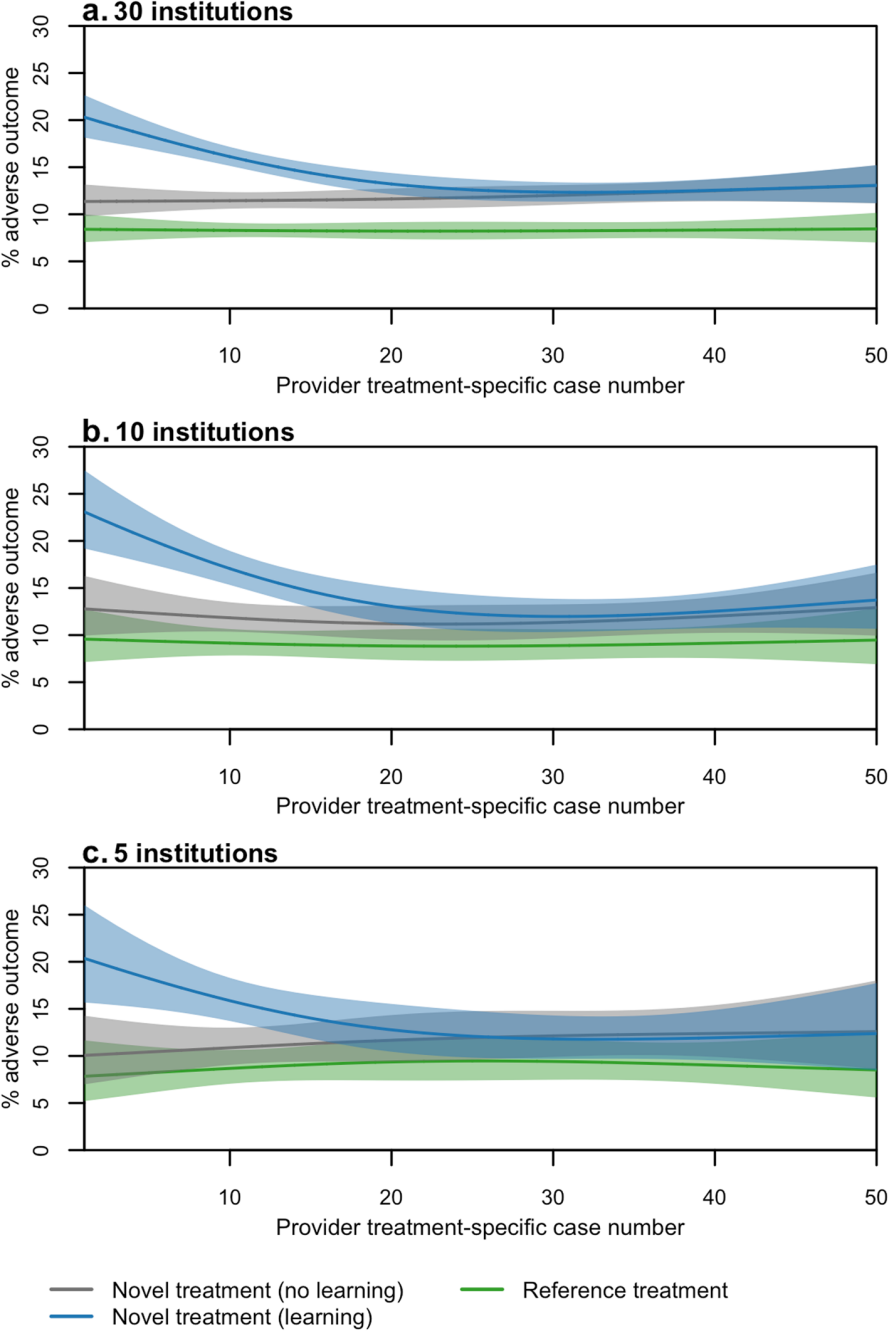
Adverse outcome rate in the presence of treatment effect and learning with varying sample sizes. Curves show smoothed probability (and 95% confidence intervals with corresponding shaded regions) of an adverse outcome over the case series. Provider learning-associated risk was specified as starting at 50% of the mean patient-associated risk among patients assigned to the novel treatment and declined toward 0 over the provider’s first 25 cases. Number of institutions simulated was set to 30 (**a**), 10 (**b**), and 5 **c**. In each simulated dataset, 50% of the population was assigned the novel treatment, the population adverse outcome rate was 10%, and the odds ratio of an adverse outcome for the novel treatment compared to the reference treatment was 2.0

Figure 6 illustrates the impact of specifying treatment and learning effects on simulated adverse outcomes, as well as the ability to recover these specified relationships as sample size changes. The plots display smoothed spline curves fit to the simulated binary outcomes by case series order among patients assigned to the reference treatment, patients assigned to the novel treatment in the absence of learning, and patients assigned to the novel treatment in the presence of learning. In these examples, the population outcome rate was specified as 10%; the odds ratio comparing the novel and reference treatments was specified as 2.0; and provider learning, when present, was specified to occur over a provider’s first 25 cases receiving the novel treatment. With data simulated for 30 institutions (Fig. 6a), both the difference in outcome rate by treatment and the stability of the outcome rates over case order for the reference and novel treatments in the absence of learning are evident. Adding a learning effect for the novel treatment, as illustrated by the blue line in Fig. 6a, injected a downward trend in outcome rates among cases early in the series only among those patients assigned the novel treatment. The outcome rate stabilized after approximately 25 cases and was equivalent to the outcome rate observed for the novel treatment when learning was absent. When data were simulated for 10 or 5 institutions (Figs. 6b and c), reducing the number of observations, confidence intervals of the smoothed curves widened and differences by treatment were less clear. However, the pattern of decreasing and stabilizing outcome rates over the novel treatment’s case series in the presence of learning was still apparent.

## Discussion

Validating and characterizing the effectiveness of new algorithms in biomedical research often requires knowing the ground truth for data characteristics under investigation. As such knowledge of the “truth” is unavailable or difficult to ascertain in real world data, simulation studies using synthetic datasets that mimic complex clinical environments can provide insight. Existing simulation studies have not addressed programmatically changing adverse outcome risk as a function of learning by clinical providers and healthcare organizations, particularly with regards to new medical devices, treatments, or procedures. This study extends the synthetic clinical data generation literature by implementing a generalizable framework for injection of hierarchical learning effects within a robust data generation process, while still accounting for complex clinical relationships.

Our DGP can enable simulation studies across clinical domains and treatment types, however, prospective post-marketing surveillance of novel medical devices, a priority of the FDA, [32] provides a grounding use case. Studies suggest that adverse events attributable to provider learning of newly approved cardiovascular devices are responsible for 50–70% of all adverse events, representing significant harm in 1–6% of patients treated [30, 31, 50–53]. For example, after the initial launch of the Cordis Cypher sirolimus-eluting coronary stent, safety questions emerged from reports of subacute thrombosis and death following stent placement. Further investigation, however, revealed that some providers were not adhering to device labeling recommendations. As a result, FDA was able to address the situation by reminding providers to follow stent size and use recommendations rather than recalling a successful, life-saving technology [54]. As this example highlights, it is critically important that post-market surveillance and comparative safety evaluations correctly attribute the source of risks associated with new medical devices to properly marshal regulatory, health system, and manufacturer resources in response safety and experiential learning concerns. New algorithms that can disentangle safety signals from learning effects could promote uptake and maintain access to safe and effective new treatments, while also generating evidence to advocate for programs to enhance learning by providers or institutions. Our DGP directly supports this needed methodological research by enabling iterative algorithm improvement and benchmarking to compare approaches.

Our DGP implementation included key assumptions of independence that should be carefully considered. We assume the probability of an adverse event due to provider learning, the probability due institutional learning, and the probability due patient characteristics are each independent from one another. This assumption simplifies the combination of these probabilities while ensuring each patients’ final probability of an adverse outcomes remains valid between 0 and 1. While this simplifies the math, we can imagine scenarios where these probabilities may not be easily separable. The pace of learning may be slower when treating patients with certain complicating factors than when treating the general population. Institutional policies, practices, and resources may influence the pace at which different providers are able to master a new treatment. In such cases, evaluations of new algorithms with simulations based on the independence assumption may prove optimistic when the algorithms are applied in real world data. Users with particular concern may consider testing the influence of our independence assumption for their use case by varying the learning effects based on simulated patient traits or across different providers.

There is also an inherent assumption in our implementation that missing values are independent of patient, provider, and institution. Missingness at random is a strong, but likely often false, assumption common in biomedical research. More likely, missing values may be informative and associated with outcomes, patient factors, and provider or institutional practice patterns. Our implementation of missingness at random, therefore, reflects a common simplification and may be a source of optimism in evaluations of novel algorithms. For use cases particularly interested in the impact of missingness, the data finalization process of our DGP would need to be extended to vary the probability of data masking by select data characteristics.

Users should also consider sample size requirements and the risk of possible deviations between realized simulations and specified parameters. Our simulation results indicated fidelity between specifications and synthetic datasets increased with sample size, especially beyond approximately 3,000 observations. This finding reflects that recovering specified relationships from the synthetic data is subject the influence of noise and uncertainty, both of which are exacerbated when estimating values with limited sample sizes. Despite increased variability when sample sizes were small, features in the synthetic datasets reflected specified settings – an observation highlight in the learning curves displayed in Fig. 6, in which the impact of learning on outcome rates was apparent even as confidence intervals widen with declining sample size. While we observed more consistent fidelity of our data above 3,000 observations, this may not reflect a rule of thumb lower bound for generating simulations responsive to specifications. Simulation complexity and characteristics of the underlying reference population likely impact simulation fidelity. For applications requiring small datasets, users may consider including an assessment of simulation fidelity as a quality check prior to evaluating new methodologies and consider multiple replicates at each parameter setting to capture and characterize variability.

This work builds on the growing literature around synthetic clinical data generation. The generalizable and flexible nature of our DGP framework allows users to extend and customize components of the process to meet their unique requirements. Users can specify complex interactions among patient features if desired, both for treatment and outcome assignment. We implemented the DGP using an iterative algorithm designed to simulate non-normal, correlated data with varying variable formats [43]. However, users could replace this module of the DGP with any state-of-the-art synthetic patient feature generator that produces a patient-level dataset. Our DGP could accept such a data cube and merge with provider and institution assignments before continuing with the DGP components specifying treatment assignment, learning effects, and outcomes.

Future improvements to the DGP described here can address additional limitations. Provider or institution-level features, such as number of years of provider clinical experience or type of institution, may be important for some methodological investigations. While the DGP does not currently support features at the provider or institutional levels, this functionality can be readily developed in future iterations through extension of components currently processing patient-level characteristics. Second, we assign each provider to a single institution, however, in practice providers often treat patients at multiple facilities. Reassigning a portion of a provider’s observations to a different institution prior to the assignment of case orders would allow provider case series to remain intact and incorporate the provider into the case series of multiple institutions. Third, while we know patient clinical profiles impact where and from which provider they receive care, the DGP currently randomizes patients to providers and institutions. Supporting non-random assignment of patients based on provider and institutional traits is a complex undertaking that we will explore in future work.

Finally, we acknowledge that no synthetic dataset can fully represent the complexity and uncertainty of real-world data. Simulation studies with their inherent simplifications, whether based on our DGP and other simulation approaches, provide an important initial validation of new algorithms and approaches. However, users should consider whether and which simplifying assumptions are acceptable for algorithmic benchmarking and anticipate some reductions in performance when methods are subsequently evaluated in case studies using clinical data.

## Conclusions

Complex simulation studies are required to develop and test new algorithms that disentangle safety signals for medical treatments from the effects of experiential learning. In support of such studies, our data generating process extends clinical data simulation techniques beyond generation of patient features to incorporate injection of hierarchical learning effects at the provider and institutional levels. Our approach has the potential to be adopted as a common resource for the scientific community to increase the efficiency of simulation studies in methods development research. In turn, supporting algorithmic validations across a range of clinical scenarios to provide guidance on the use and interpretation of new methods and algorithms. Once validated in synthetic data, those methods that correctly attribute treatment and learning risk can be applied to real world data to identify training opportunities to accelerate optimal performance and avoid unwarranted restriction of patients’ access to medical advances.

## Supplementary Information

**Additional file 1: Appendix Table A.1.** Full description of available specification parameters.** Appendix Table A.2.** Implemented function learning curve forms where $x$ is the case number, $b$ is the initial learning-associated probability of an adverse outcome. Other parameter values are calculated based on the specified speed of learning.** Appendix Figure A.1.** Illustrative learning curves for the forms available in the current DGP implementation. Example curves shows a situation in which learning occurs over 100 cases and reduces reducing initial risk by 25% for a device with a steady state outcome rate of 10%.** Appendix Figure A.4.** For each patient-level feature, distribution of mean values across simulated datasets grouped by ranges of sample size.** Appendix Figure A.5.** For each continuous patient-level feature, distribution of standard deviation values across simulated datasets grouped by ranges of sample size.

## Data Availability

The data that support the findings of this study are available from the MIMIC project and made available through the MIT Laboratory for Computational Physiology. These data are publicly available at https://mimic.mit.edu/ from the original distributor through data use agreements.

## Abbreviations

DGP: Data generating process
FDA: U.S. Food and Drug Administration
