# Supplementary material for: Simulating complex patient populations with hierarchical learning effects to support methods development for post-market surveillance
Source: BMC Med Res Methodol. 2023 Apr 11;23:89. doi: 10.1186/s12874-023-01913-9 (PMC10088292; doi:10.1186/s12874-023-01913-9)

**Appendix Table A.1.** Full description of available specification parameters.

| **Feature** | **Description** | **Details/Options** |
| --- | --- | --- |
| # of institutions | Total number of institutions to represent in the dataset |  |
| Operator distribution | Distribution of number of operators within each institution | Options: specified number, truncated normal distribution, bimodal mixture of normal distributions |
| Patient feature set | Distributions and correlations of patient features | Option 1: Users may specify a distribution for each desired patient feature (based on registry or published data summaries). Patient features will be independent or correlated based on user-specified correlation matrix.  Option 2: Users may provide an EHR-derived dataset with patient features. Data will be generated to reflect any complex, non-normal distributions and the correlation structure in the original input data. |
| Operator case volumes | Distribution of the annual number of patients treated by an operator. | This can be bimodal to reflect the presence of high and low volume providers.  Options: specified number, truncated normal distribution, bimodal mixture of normal distributions |
| Operator entry | Timing of operator entry into case series – single or annual entry | Under annual entry, 50% of operators at each institution have cases from the start, with additional operators entering the case series each year. |
| Device prevalence | Proportion of patients receiving the device of interest |  |
| Device associations | Associations specifying how patient features influence device selection | Odds ratios specifying association between assignment to the device of interest and all patient features (including any interactions). Use 1.0 to create uninformative features. |
| Device safety signal | Difference in the risk of an adverse outcome associated with the device of interest | Odds ratio for device of interest when patient features are accounted for. |
| Outcome risk factors | Associations specifying how patient features influence the risk of an adverse outcome | Odds ratios specifying association between outcome and all patient features (including any interactions). Use 1.0 to create uninformative features. |
| Adverse outcome rate | Proportion of population experiencing an adverse outcome at steady state |  |
| Operator learning – presence | Indicator of whether learning effects for the device of interest should be generated at the operator level |  |
| Operator learning - form | Functional form of learning effect | Impacts the steepness or steadiness of the learning process.  Options: Predefined forms include power, exponential, reciprocal, and Weibull. Users may define additional forms.  Ignored if operator learning absent. |
| Operator learning - speed | Number of cases with device of interest for operator to reach 95% of asymptotic performance | Ignored if operator learning absent. |
| Operator learning - magnitude | Magnitude of reduction in adverse outcome rate from device introduction to operator steady state (i.e., all learning achieved) | Specify as proportion of device of interest’s steady state adverse outcome rate. For example: A 25% reduction for a device with an initial outcome rate of 20% would result in a device with a 15% outcome rate at steady state (post learning).  Ignored if operator learning absent. |
| Institutional learning – presence | Indicator of whether learning effects for the device of interest should be generated at the institution level |  |
| Institutional learning - form | Functional form of learning effect | Impacts the steepness or steadiness of the learning process.  Options: Predefined forms include power, exponential, reciprocal, and Weibull. Users may define additional forms.  Ignored if institutional learning absent. |
| Institutional learning - speed | Number of cases with device of interest for institution to reach 95% of asymptotic performance | Ignored if institutional learning absent. |
| Institutional learning - magnitude | Magnitude of reduction in adverse outcome rate from device introduction to institutional steady state (i.e., all learning achieved) | Specify as proportion of device of interest’s steady state adverse outcome rate. For example: A 25% reduction for a device with an initial outcome rate of 20% would result in a device with a 15% outcome rate at steady state (post learning).  Ignored if institutional learning absent. |
| Missingness | Proportion of missing values across all patient features | Implemented as missing at random. |
| Noise | Additional random noise in outcome generation | In addition to noise created by the conversion of probabilities to binary outcomes, users may specify more variance in outcomes as % of outcomes to be reassigned using the steady state event rate. |
| Omitted variables | List of variables to be excluded from final output | This allows patient features that may be informative of device or outcome assignment to be masked from users |

**Appendix Description 1.** Details of DGP process, variables, and equations.

**Variables:**

- $n_{inst}$: number of institutions
- $n_{prov, i}$: number of providers at the *i*th institution
- ${Prov}_{i,j}$: *j*th provider at the *i*th institution
- $n_{pt,i,j}$: number of patients treated by provider ${Prov}_{i,j}$ each year
- $t:$ number of years of patients to generate
- $n_{pt}$: number of patients in dataset
- $p_{t}:$ proportion of patients to receive the novel treatment
- $p_{y}:$ proportion of patients to experience an adverse outcome based on patient features and treatment only
- $\boldsymbol{X}_{k,i,j}$: patient-level features for the *k*th patient treated by the *j*th provider at the *i*th institution
- ${p_{trt}}_{k,i,j}$: probability of receiving the novel treatment for the *k*th patient treated by the *j*th provider at the *i*th institution
- $\boldsymbol{\beta}_{trt}$: vector of log odds ratios of receiving the novel vs reference treatment
- ${trt}_{k,i,j}$: 0/1 indicator of novel treatment assigned to the *k*th patient treated by the *j*th provider at the *i*th institution
- ${CN}_{prov}$: patient’s number in their assigned provider’s case series
- ${CN}_{inst}$: patient’s number in their assigned institution case series
- $\boldsymbol{\beta}_{y}$: vector of log odds ratios of an adverse outcome associated with patient features
- $\beta_{signal}$: log odds ratios of an adverse outcome associated with the novel treatment’s safety signal
- ${p_{pt}}_{k,i,j}$: probability of adverse outcome associated with patient features and treatment for the *k*th patient treated by the *j*th provider at the *i*th institution
- ${CN}_{prov}$: patient’s case number in the associated provider’s ordered case series
- ${CN}_{inst}$: patient’s case number in the associated institution’s ordered case series
- $f\left( {CN}_{prov} \right)$: functional form of provider learning curve
- $f\left( {CN}_{inst} \right)$: functional form of institutional learning curve
- ${p_{prov}}_{k,i,j}$: probability of adverse outcome associated with patient features and treatment for the *k*th patient treated by the *j*th provider at the *i*th institution
- ${p_{inst}}_{k,i,j}$: probability of adverse outcome associated with patient features and treatment for the *k*th patient treated by the *j*th provider at the *i*th institution
- $p_{k,i,j}$: probability of an adverse outcome assigned to the *k*th patient treated by the *j*th provider at the *i*th institution taking into account all specified effects
- $y_{k,i,j}$: 0/1 indicator of an adverse outcome assigned to the *k*th patient treated by the *j*th provider at the *i*th institution
- $p_{MAR}$: proportion of all patient features values to be masked
- $\boldsymbol{X}_{omit}$: vector of patient features to be omitted from final dataset

**Steps:**

1. Patient Generation
   1. For each of $n_{inst}$ institutions, generate $n_{prov, i}$ from a user-specified distribution or mixture of distributions of the number of providers per institution.
   2. For each ${Prov}_{i,j}$, generate $n_{pt,i,j}$ from a user-specified distribution or mixture of distributions of the number of patients treated annually by a provider.
   3. Assign each provider to be included in the case series for 1 to $t$ years.
   4. For each ${Prov}_{i,j}$, generate patients records based on the number of years assigned and $n_{pt,i,j}$. Randomly order the patients and assign each to a particular year based on $n_{pt,i,j}$.
   5. Assign features ($X_{k,i,j}$) for each patient record based on user-specified approach.
2. Treatment assignment
   1. Calculate probability of receiving the novel treatment as

$${p_{trt}}_{k,i,j}=e^{\alpha+B_{trt}X_{k,i,j}} / (1+e^{{\alpha+B}_{trt}X_{k,i,j}})$$

where $\alpha$ is estimated to adjust the novel treatment frequency to

- 1. Assign indicator of novel vs reference treatment as

$${trt}_{k,i,j}=Bernoulli({p_{trt}}_{k,i,j})$$

1. Case ordering
   1. Randomly order all patients within assigned year
   2. Using this ordering, institution-specific case order (${CN}_{inst})$
   3. Using this ordering, assign provider-specific case order (${CN}_{prov})$
2. Outcome assignment
   1. Calculate probability of adverse outcomes due to patient features and treatment as

$${p_{pt}}_{k,i,j}=e^{{\alpha+\boldsymbol{\beta}}_{y}\boldsymbol{X}_{k,i,j}+\beta_{signal}{trt}_{k,i,j}} / (1+e^{{\alpha+\boldsymbol{\beta}}_{y}\boldsymbol{X}_{k,i,j}+\beta_{signal}{trt}_{k,i,j}})$$

where $\alpha$ is estimated to adjust the adverse outcome rate to

- 1. Calculate probability of adverse outcomes due to provider learning based on specified learning function as
  ${p_{prov}}_{k,i,j}=f\left( {CN}_{prov} \right)$
  2. Calculate probability of adverse outcomes due to institutional learning based on specified learning function as
     ${p_{prov}}_{k,i,j}=f({CN}_{inst})$
  3. Calculate joint probability of no adverse event as
     $p_{k,i,j}(y=0)=\left( 1-p_{{pt}_{k,i,j}} \right)*\left( 1-p_{{prov}_{k,i,j}} \right)*\left( 1-p_{{inst}_{k,i,j}} \right)$
  4. Calculate probability of an adverse event as
     $p_{k,i,j}\left( y=1 \right)=1-p_{k,i,j}(y=0)$
  5. Assign indicator of an adverse outcome as

$$y_{k,i,j}=Bernoulli({p_{pt}}_{k,i,j})$$

1. Finalize data
   1. Add random noise by randomly sampling $n_{pt}*p_{noise}$ patients and flipping their assigned $y_{k,i,j}$
   2. Inject missingness at random by randomly selecting $p_{MAR}$ values across
   3. Create omitted variables by dropping all patient features in $\boldsymbol{X}_{omit}$

**Appendix Table A.2.** Implemented function learning curve forms where $x$ is the case number, $b$ is the initial learning-associated probability of an adverse outcome. Other parameter values are calculated based on the specified speed of learning.

| **Form** | **Equation** |
| --- | --- |
| Power | ${bx}^{-c}$ |
| Exponential | $b/x$ |
| Reciprocal | $b*exp(-cx)$ |
| Weibull | $b*exp(-cx^{d})$ |

**Appendix Figure A.1.** Illustrative learning curves for the forms available in the current DGP implementation. Example curves shows a situation in which learning occurs over 100 cases and reduces reducing initial risk by 25% for a device with a steady state outcome rate of 10%.


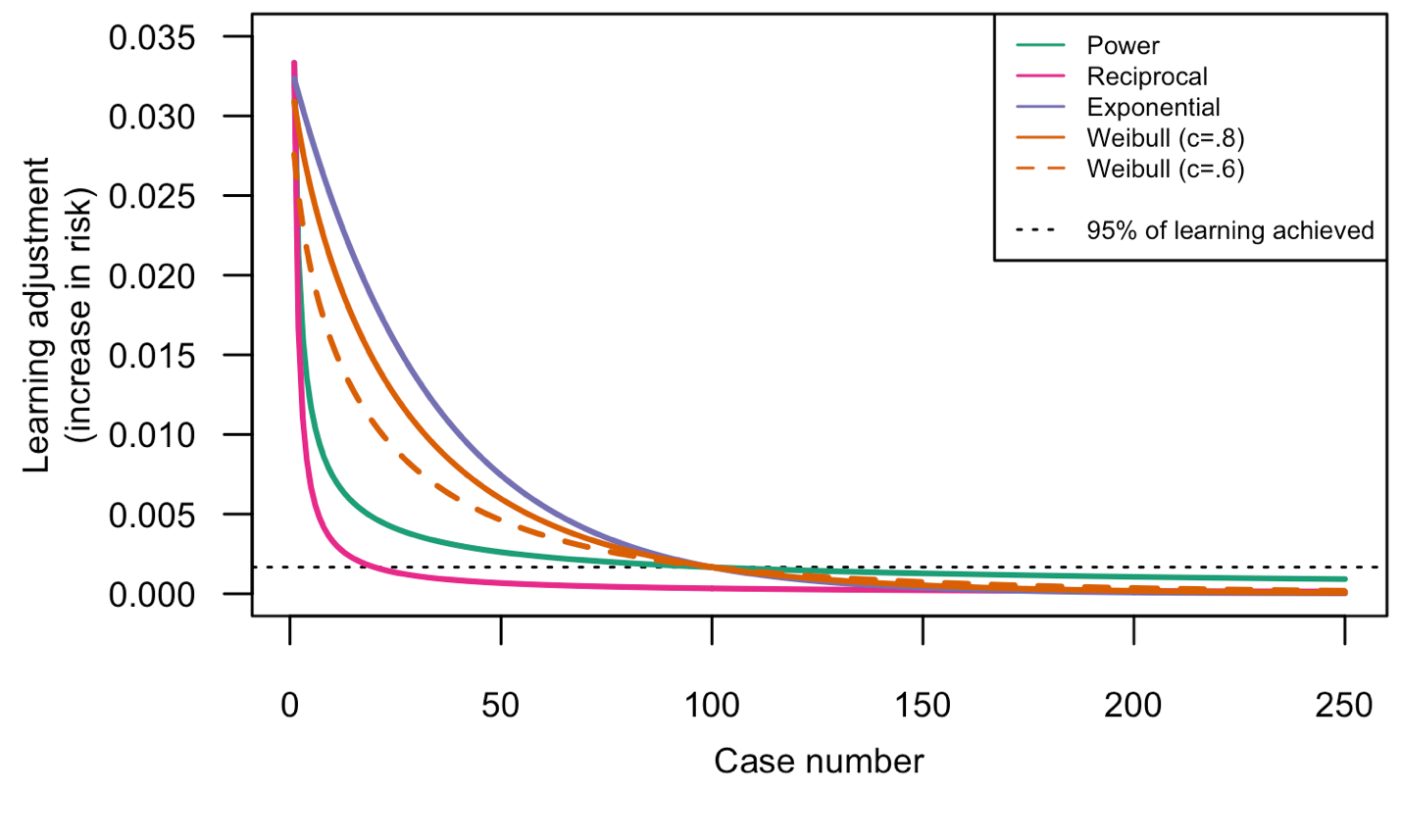


**Appendix Description 2.** Details of all DGP options specified in the simulation.

Underlying population – EHR datacube – MIMIC III aline population

Device options

- Device of interest – Device B in all datasets
- Prevalence – Prevalence of device B set to 10%, 25%, or 50%
- Feature associations – Associations between patient features and device assignment randomly selected from a “population” of 5 association set ups.
- Safety signal – Odds ratio for outcome associated with device B set to 1.0, 1.5, 2.0

Institutions

- Number – 5, 10, 20 or 30
- Operator distribution – Distribution of the number of operators at each institution will be a mixture model with 50% high volume (10 providers) and 50% low volume (5 providers)
- Learning effects – For device B, combinations of each of the following:
  - Presence and absence
  - Form: power, exponential, or Weibull
  - Magnitude:5%, 10%, 25%
  - Speed: 100 or 200 cases to reach 95% of steady state

Operators

- Case volumes – Distribution of the annual number of patients treated by an operator will be a mixture model with 50% high volume (mean 25 patients, range 20-30 and sd=2) and 50% low volume (mean 10 patients, range 5-15 and sd=2).
- Entry timing – Annual entry. 50% of each institution’s operators begin at the start of the case series, with the remainder divided even to begin their case series at each new year.
- Learning effects – For device B, combinations of each of the following:
  - Presence and absence
  - Form: power, exponential, or Weibull
  - Magnitude: 20%, 40%, 60%
  - Speed: 10 or 25 cases to reach 95% of steady state

Outcome

- Feature associations – Associations between patient features and adverse outcomes randomly selected from a “population” of 5 association sets
- Overall event rate – 2%, 5%, 10%
- Noise – No extra noise beyond binomial outcome generation from probabilities.

Other

- Missingness – 0%, 5%, 10% -- not generated separately, on each population with 0 missingness, also document device signal estimate after adding missingness
- Omitted variables – None.
- Timeframe – 2 or 4 years of cases

**Appendix Figure A.4** For each patient-level feature, distribution of mean values across simulated datasets grouped by ranges of sample size.
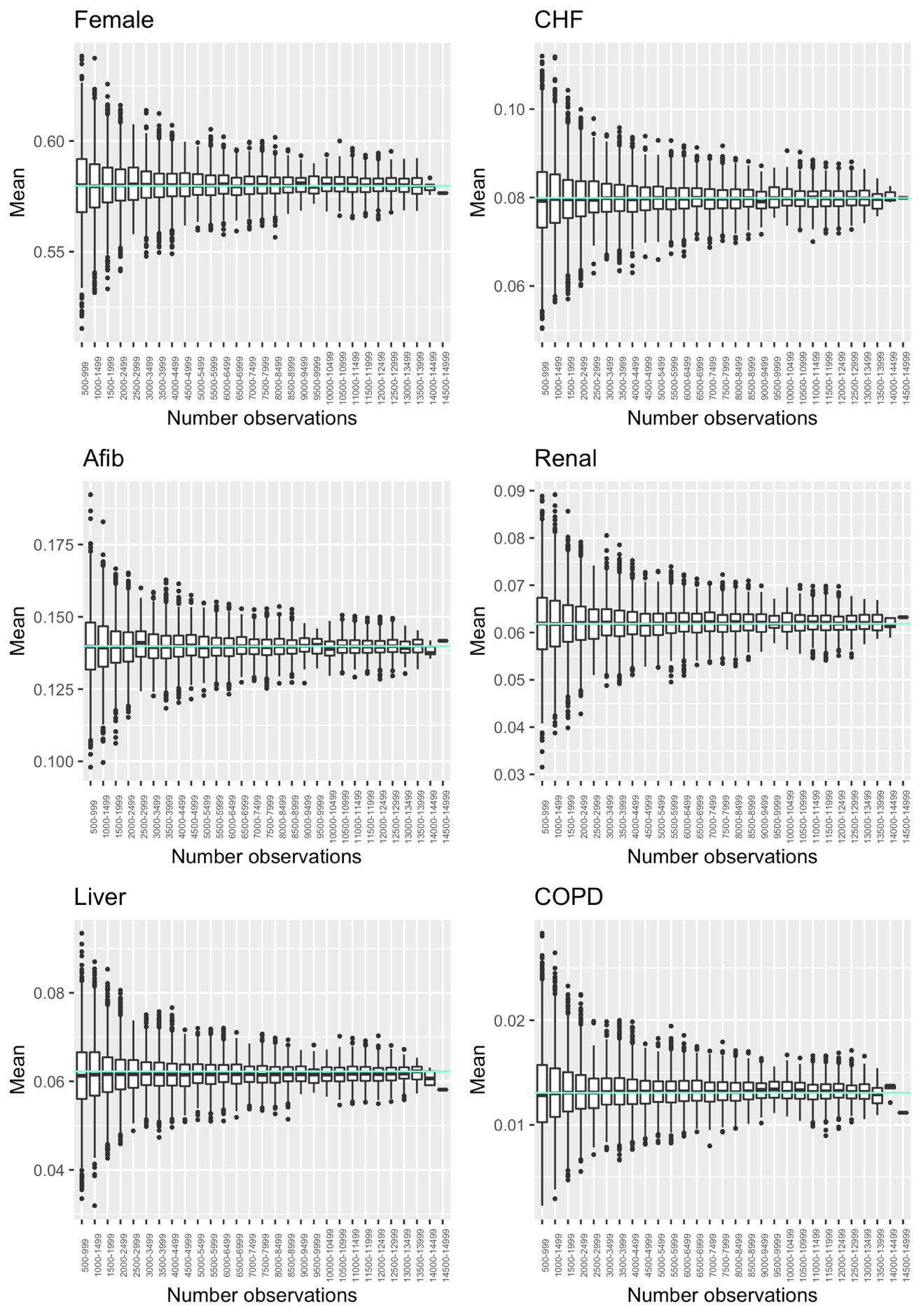


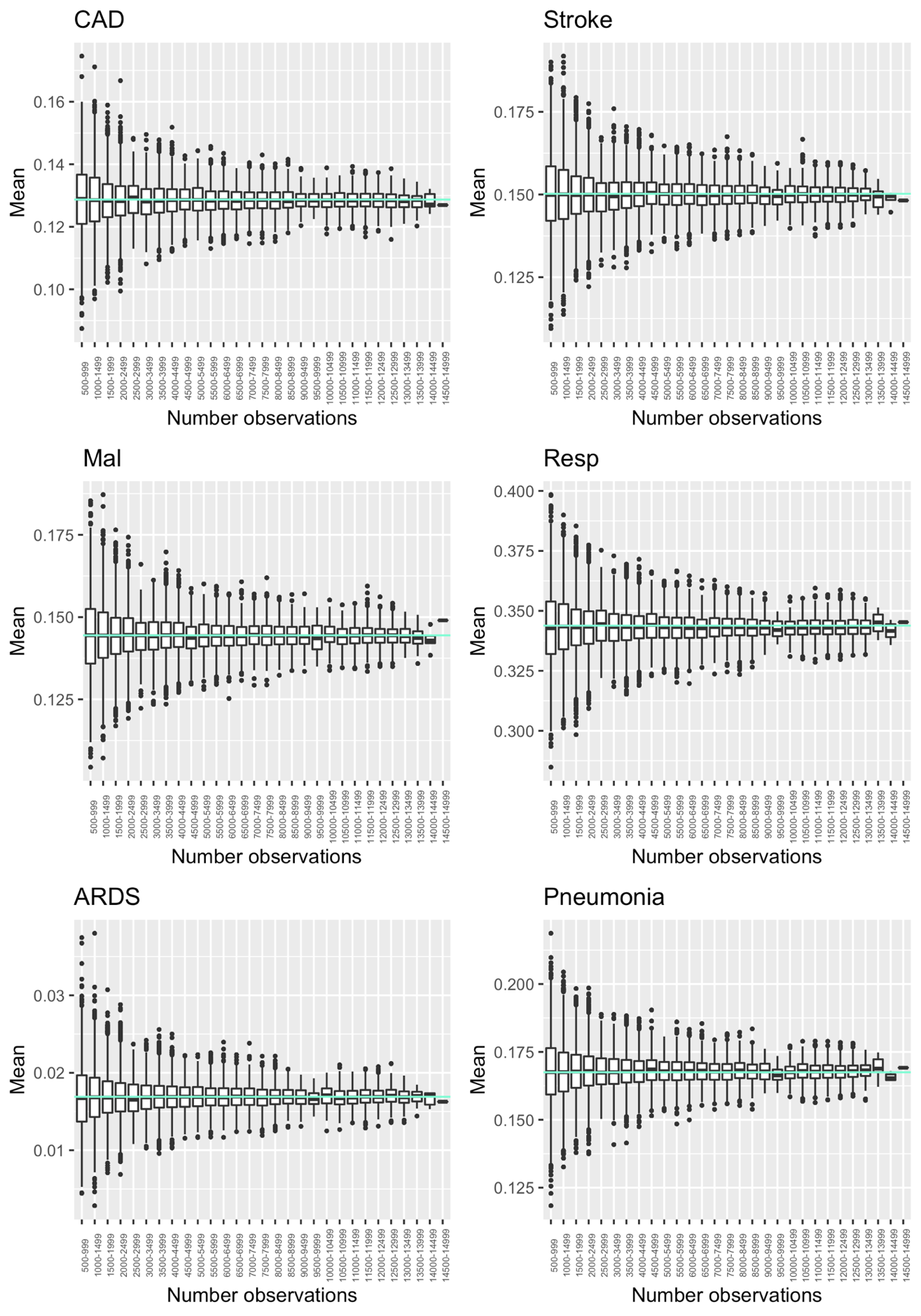

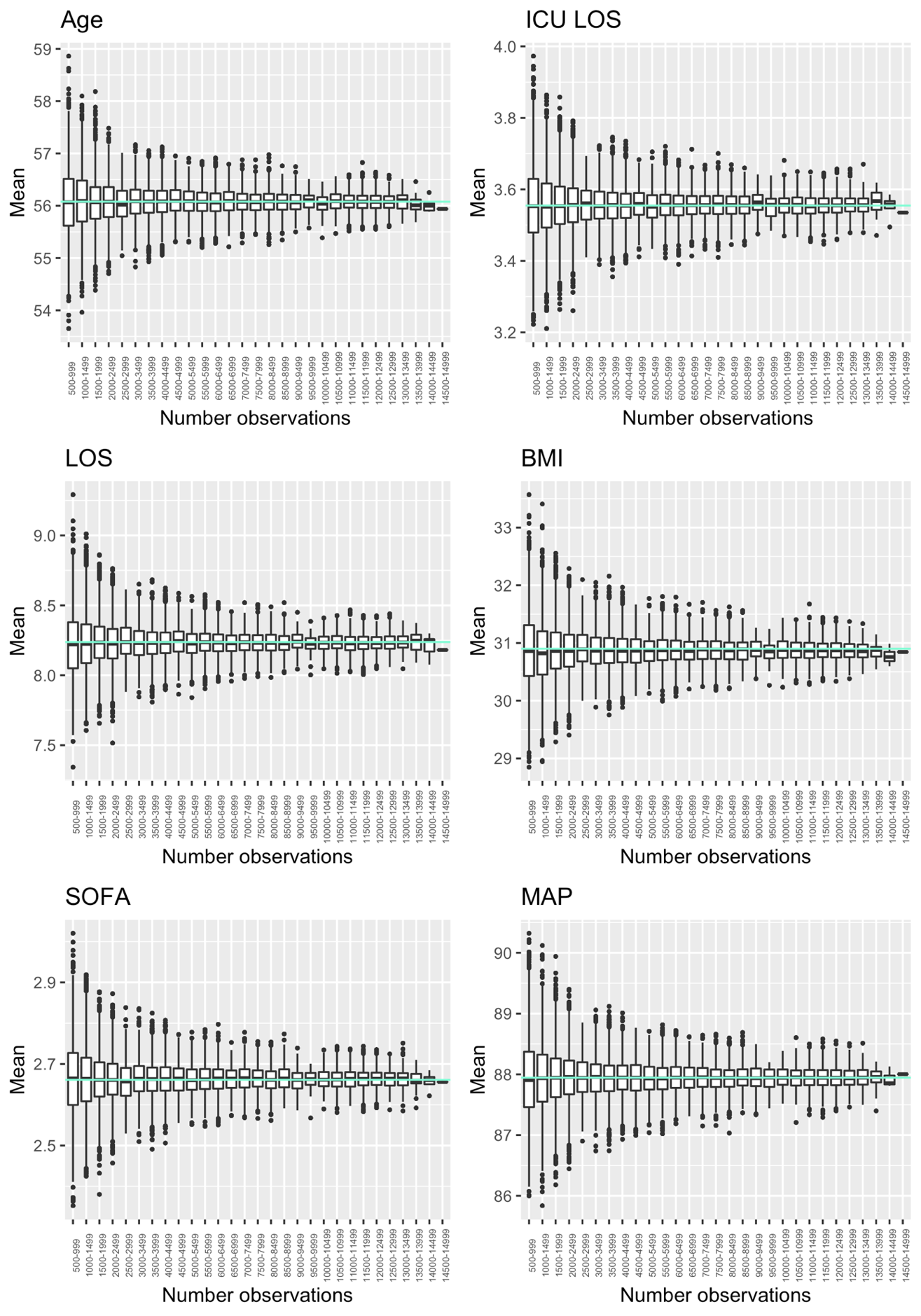

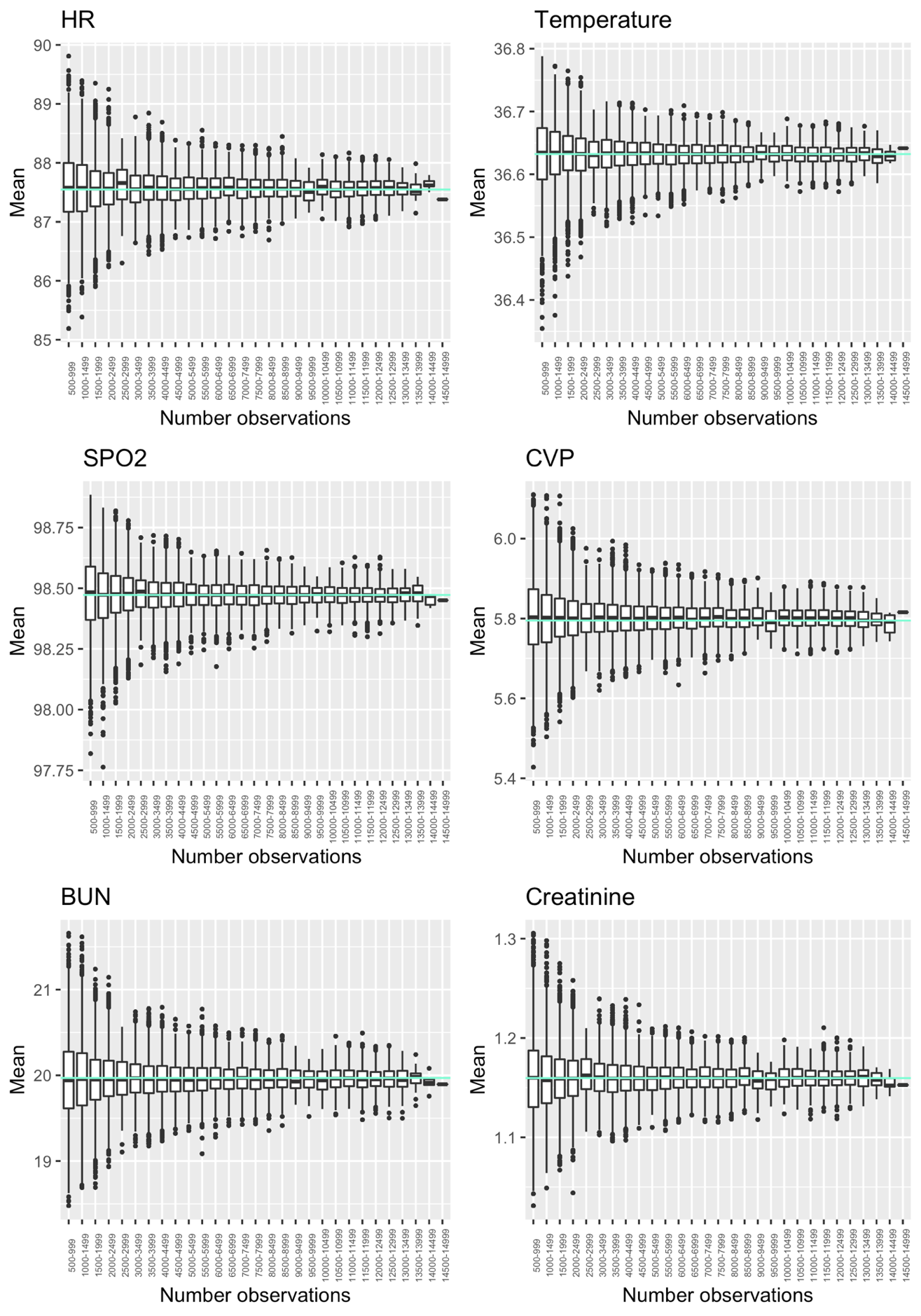

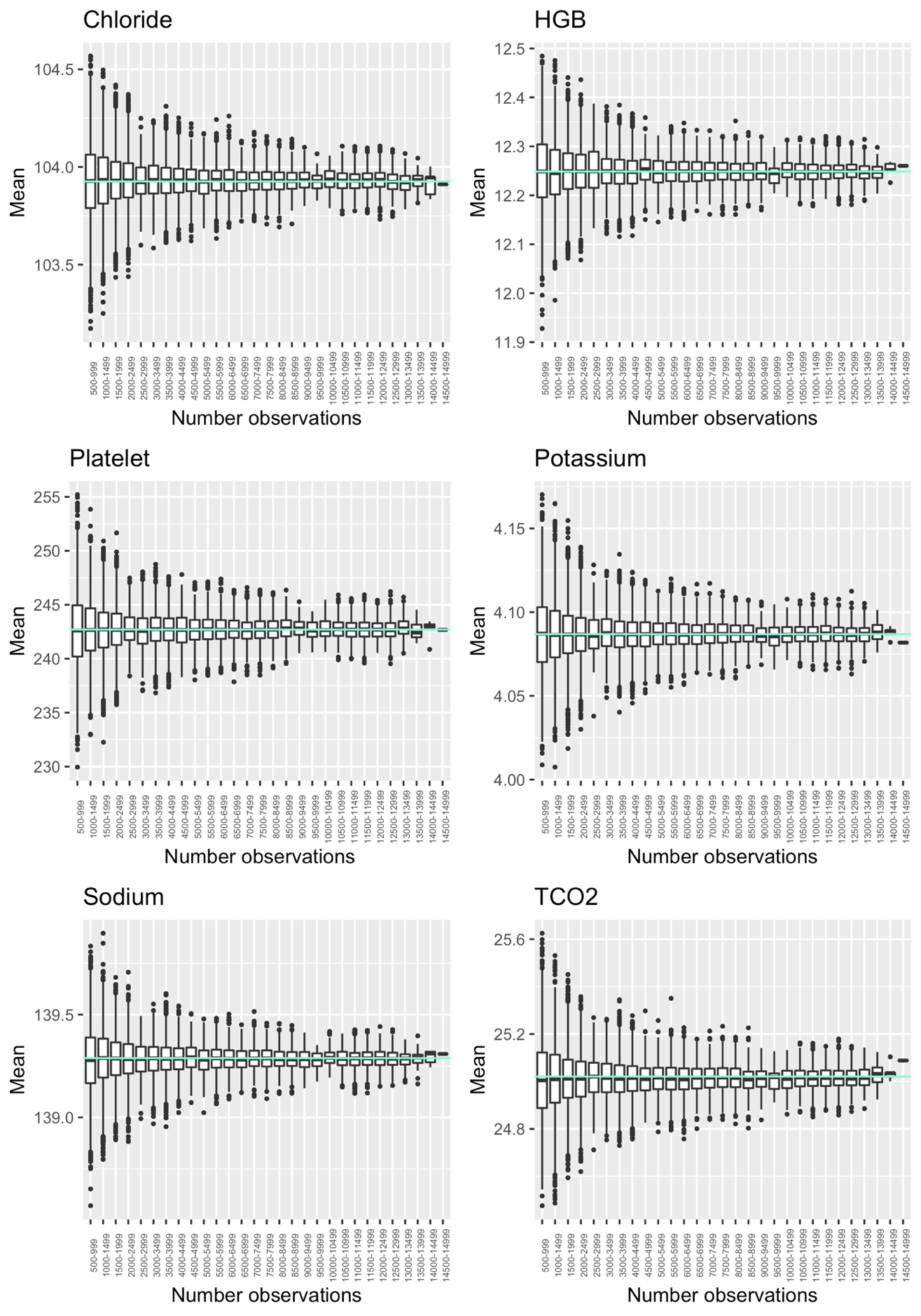


**Appendix Figure A.5** For each continuous patient-level feature, distribution of standard deviation values across simulated datasets grouped by ranges of sample size.


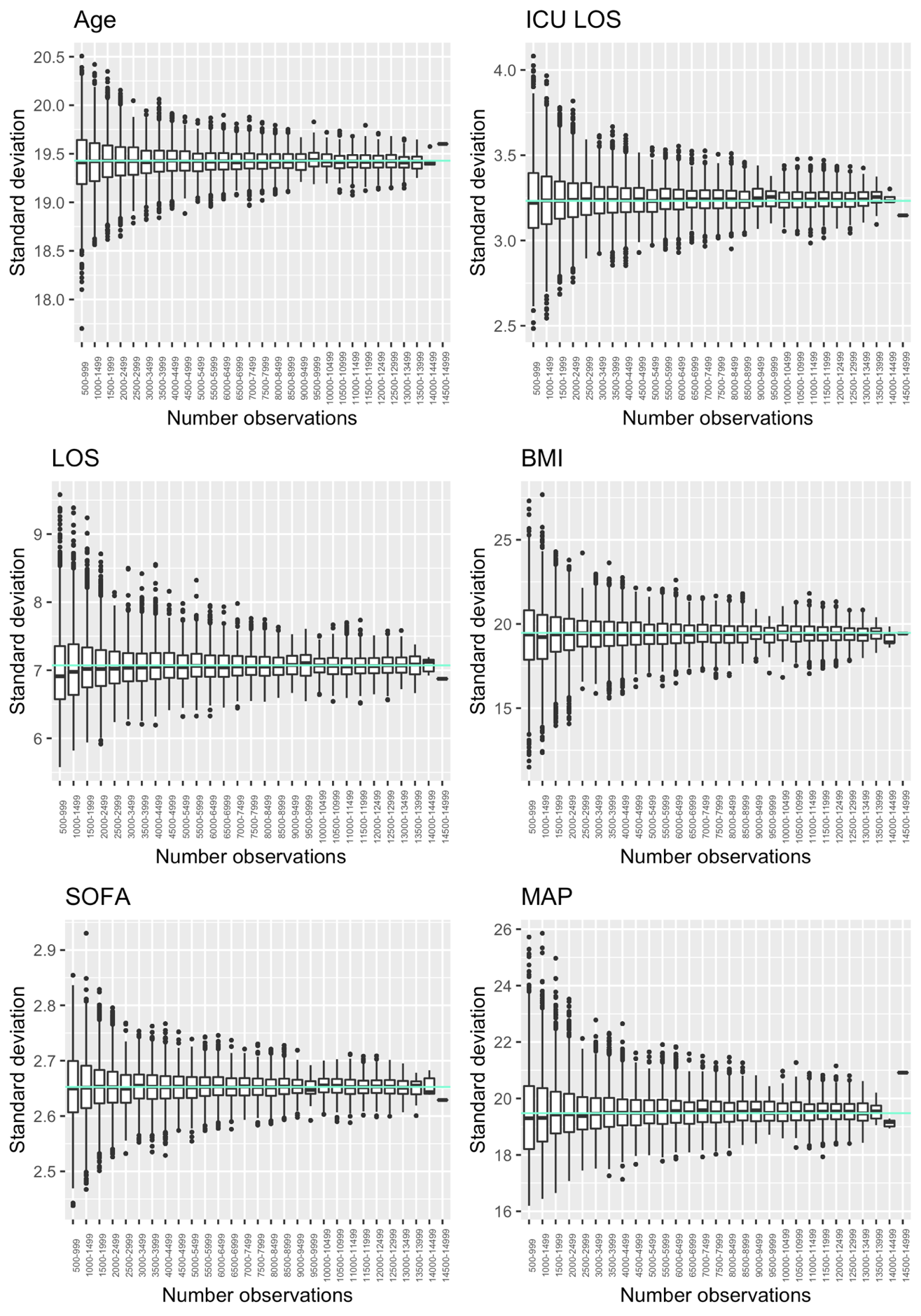


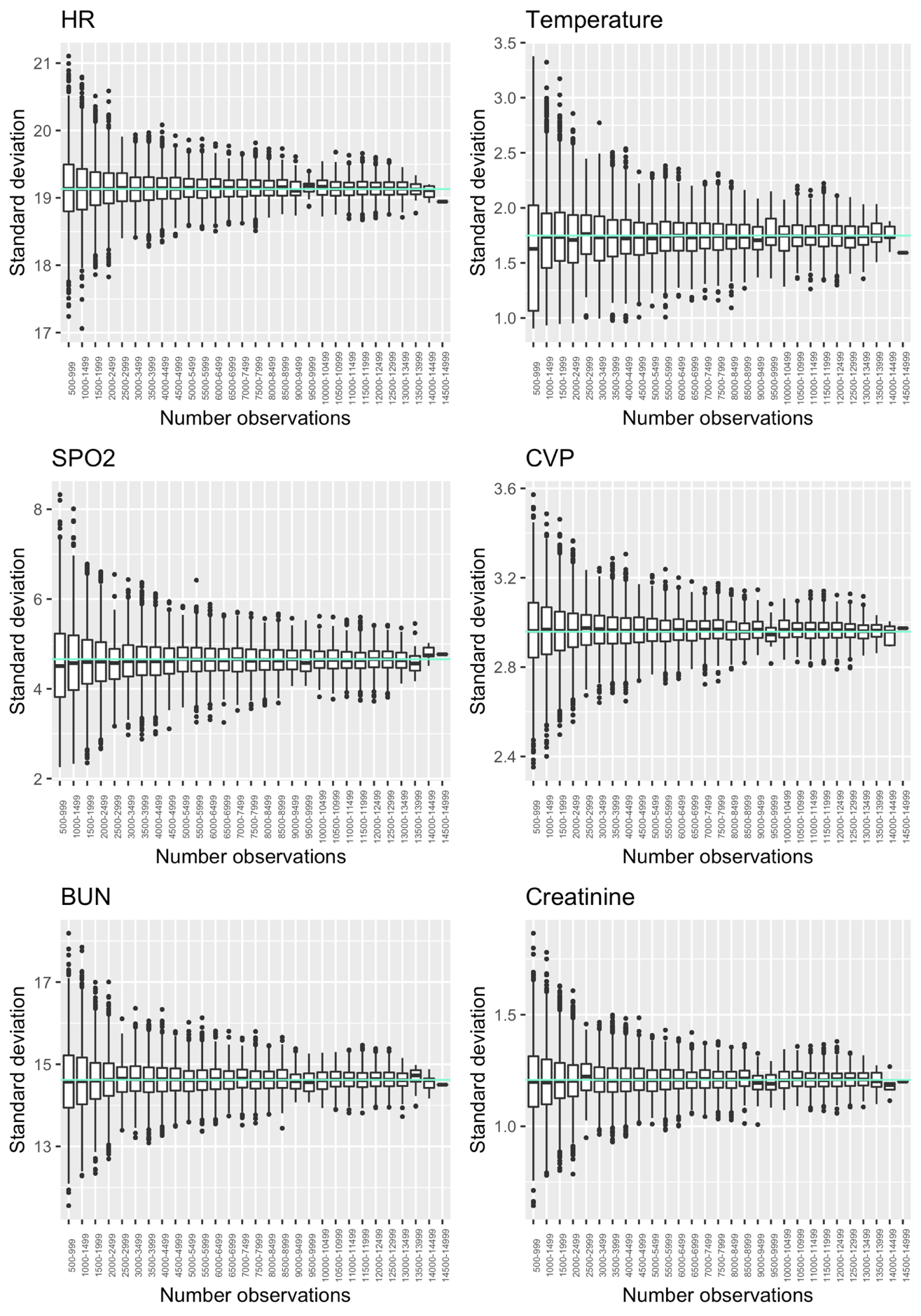

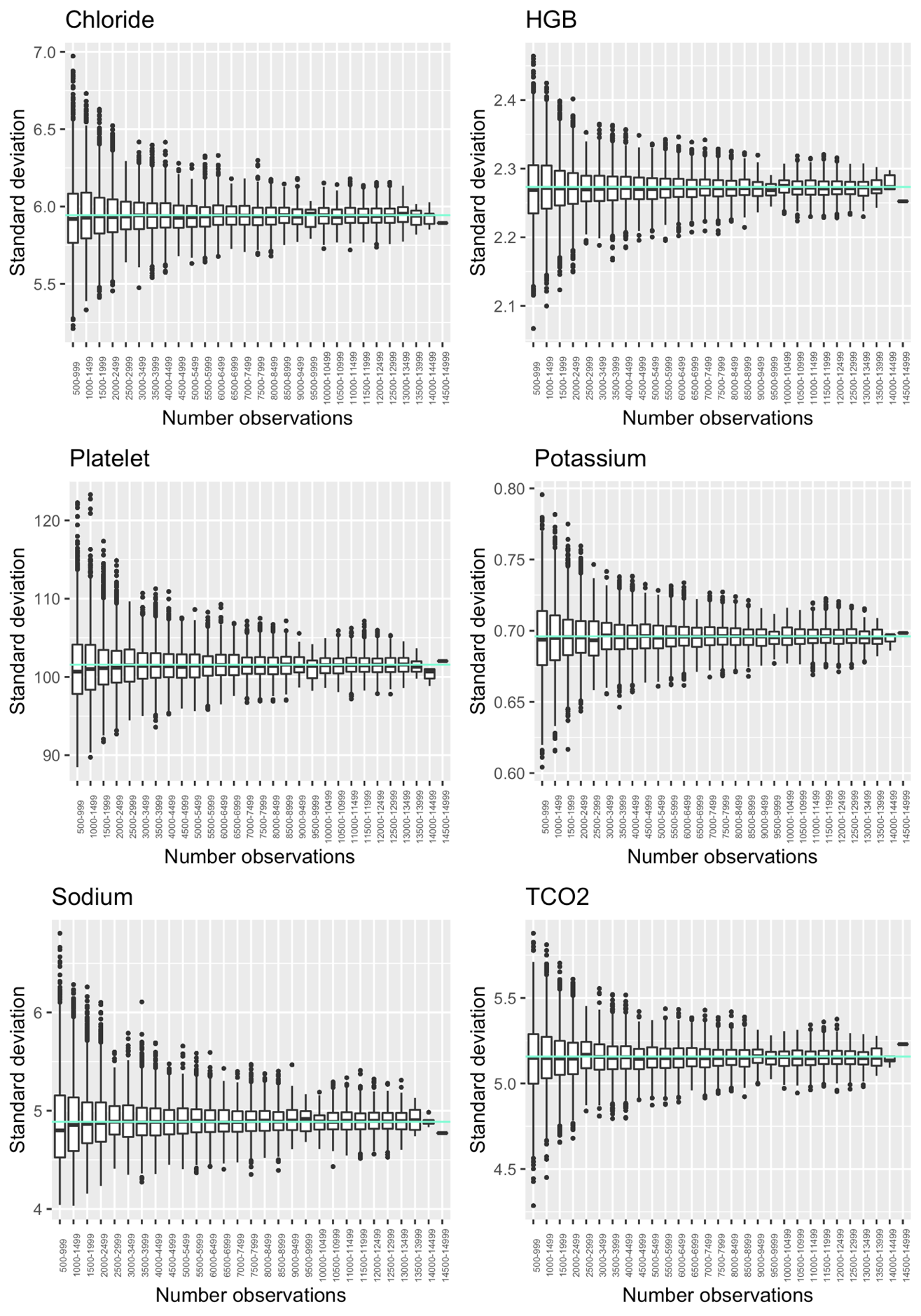

Supplement: Supplementary file 1 — Additional file 1: Appendix Table A.1. Full description of available specification parameters. Appendix Table A.2. Implemented function learning curve forms where $x$x is the case number, $b$b is the initial learning-associated probability of an adverse outcome. Other parameter values are calculated based on the specified speed of learning. Appendix Figure A.1. Illustrative learning curves for the forms available in the current DGP implementation. Example curves shows a situation in which learning occurs over 100 cases and reduces reducing initial risk by 25% for a device with a steady state outcome rate of 10%. Appendix Figure A.4. For each patient-level feature, distribution of mean values across simulated datasets grouped by ranges of sample size. Appendix Figure A.5. For each continuous patient-level feature, distribution of standard deviation values across simulated datasets grouped by ranges of sample size. [file 12874_2023_1913_MOESM1_ESM.docx]
